# Functional trait dataset of European groundwater Amphipoda: Niphargidae and Typhlogammaridae

**DOI:** 10.1038/s41597-024-03020-w

**Published:** 2024-02-10

**Authors:** Ester Premate, Cene Fišer

**Affiliations:** University of Ljubljana, Biotechnical Faculty, Department of Biology, SubBioLab, Jamnikarjeva 101, SI-1000 Ljubljana, Slovenia

**Keywords:** Zoology, Biodiversity, Freshwater ecology

## Abstract

Groundwater represents a vast, but mostly hidden and inaccessible ecosystem. Although often overlooked in freshwater research, groundwater organisms form a significant part of freshwater biodiversity, whereas their functions are crucial in different ecosystem processes. Knowledge on functional traits is generally lacking for most groundwater species worldwide, yet European groundwater amphipods, particularly the family Niphargidae, are an exception. They are well-researched and used as a model system in ecological and evolutionary studies. We focused on this group to assemble a first functional trait dataset dedicated to groundwater species. We gathered data for eight morphological functional traits quantified through 27 measurements for 1123 individuals which represent 180 species and 314 MOTUs. Besides functional trait data, every entry is accompanied with locality information, including habitat type, and DNA sequences if available. The structure of the dataset and data processing information provided along enable wide applicability and extension to other amphipod taxa. When coupled with phylogeny, the dataset may further enhance different aspects of groundwater research, including biodiversity patterns, community assembly processes, and trait evolution.

## Background & Summary

Functional traits have become an important tool in basic and applied ecological research. Among other, they provide an insight into functional roles of organisms in their environments, diversity of functions and ecological processes within ecosystems, and community response to environmental change^1–3^. In the era of the broad usage of functional traits in ecology, the constant production of trait data across different taxa and ecosystems prompted the organization of these data in publicly available datasets and databases. In this respect, freshwater organisms and ecosystems received a considerable amount of attention worldwide^4–7^. Yet, freshwater’s hidden realm, the groundwater, has been overlooked, and functional traits of groundwater invertebrates largely unexplored compared to their epigean counterparts^8–10^.

Groundwater represents a vast, globally widespread ecosystem which stores by far the largest portion of available freshwater^11–14^. Groundwater invertebrates significantly contribute to the overall biodiversity of freshwater ecosystems^15^ and play vital roles in ecosystem processes such as nutrient cycling and bioturbation. Groundwater provides a number of ecosystem services, from supporting terrestrial and epigean freshwater ecosystems to the provision of drinking water^12^, all of which depend on the functional traits of groundwater biota. Exploration of functional traits and gathering trait data of groundwater invertebrates is thus critical for the advancement in our understanding of groundwater ecological processes, ecosystem services, and responses to environmental change^8,9,16,17^.

Functional trait data for few groundwater invertebrate species are included in publicly available databases^6,7^, but are limited to only few taxa and often incomplete. Additionally, functional trait entries of groundwater organisms are usually very general and wide to fit in the databases focusing on freshwater invertebrates, meaning that they might not carry sufficient information to study processes within the groundwater itself (e.g. the value for certain functional trait would be the same across all groundwater taxa). Recently, Hose *et al*.^8^ provided a general overview of expected functional trait states in groundwater invertebrates, but showed that available knowledge is scarce. Reasons for this knowledge gap primarily lie in habitat inaccessibility, but also in difficulties associated with rearing and studying groundwater invertebrates in laboratory conditions^18^. Both hamper *in-situ* observations and experimental testing of traits’ functionality. Nevertheless, a long history of research of groundwater amphipods paved the way to the organization of the first functional trait dataset dedicated to subterranean species.

Amphipods are an important macroinvertebrate group in groundwater, both in terms of abundances and species richness^19^. They inhabit all continents except Antarctica, and they even survived the Pleistocene glaciation in Iceland thermal waters^20^. In the Western Palearctic, amphipods comprise about one third of all subterranean crustaceans^21^. The most common and studied genus among them is *Niphargus*, which is found across different groundwater habitats. It is the largest freshwater amphipod genus^19,22^ and an important model system in ecology and evolution^23–25^. Previous laboratory and comparative studies have identified several functional traits related to habitat selection, locomotion, feeding, reproduction, and defense against predators^24–29^. Studies that explicitly tested functionality of different traits provided baseline knowledge of functional traits for groundwater amphipods. We compiled functional trait data available from already published and unpublished datasets and collected new data for missing and still undescribed species.

With the Functional trait dataset of European groundwater Amphipoda: Niphargidae and Typhlogammaridae, we aim to facilitate the use of functional trait data in groundwater research. Crustaceans are globally well-represented in groundwater communities, dominating in terms of species richness^30^. As such, they are suitable model systems for tackling various ecological and evolutionary questions through the usage of functional traits, especially when combined with phylogenetic, ecological, and biogeographical data. The present dataset combines all: individual-level trait data, ecology, geographical location, and cytochrome oxidase I (COI) gene sequences, and can thus be readily used to assess e.g. local and regional biodiversity patterns and community assembly processes, as well as to study the evolution of functional traits. At the same time, it is the first functional trait dataset of such extent and completeness for both subterranean fauna and amphipods. It serves as a basis for future publicly available online database, which will gather functional trait data for subterranean amphipods and offer the possibility to update and contribute data, as well as to extend it to other freshwater and marine amphipod taxa.

## Methods

### Selection of functional traits

Functional traits are any traits influencing organismal performance^3,31^, and can be morphological, behavioral, physiological, or life-history. Most of the trait types, except morphological, are difficult to measure in groundwater invertebrates, as their collection, rearing, or observation in their natural habitat is at least limited, if not impossible. They are also less abundant than invertebrates in other freshwater ecosystems and reaching adequate sample size is often hindered^18^. We focused on morphological traits that have been linked to a specific function in prior studies. We provide a review of these traits and their corresponding functions below and in Table 1.

**Table 1:** Overview of functional traits included in Functional trait dataset of European Groundwater Amphipoda: Niphargidae and Typhlogammaridae. Letters in the first column correspond to Fig. 1 panels where the measurements and functional traits are presented.

	Functional trait	Function - general	Function - detailed	Measurements	References
A	Body size	Complex	Affects metabolic rate, dispersal ability, locomotion, microhabitat selection. Sexually dimorphic trait in some species.	Body length	^24,32^
A, B	Body shape including ventral channel shape	Complex	Overall body shape affects locomotion and microhabitat selection. The depth of the ventral channel determines the velocity of water flow which brings oxygen to the gills and creates propulsion when moving. It also determines the maximum size of the marsupium.	Coxa II depth	^24,26,33^
				Coxa III depth	
				Pereopod V basis width	
				Pereopod VI basis width	
				Pereopod VII basis width	
A	Locomotory apparatus	Locomotion	Length of the appendages involved in locomotion relates to movement speed.	Pereopod V length	^27^
				Pereopod VI length	
				Pereopod VII length	
A	Antennae	Sensory	Antennae carry numerous chemoreceptors and are involved in chemoreception.	Antenna I length	^33^
				Antenna II length	
C	Gnathopods	Feeding	Gnathopods are used for grabbing food particles and grooming. Their size and shape relates to feeding habits and trophic position.	Gnathopod I carpus length	^28,34,35^
				Gnathopod II carpus length	
				Gnathopod I propodus length	
				Gnathopod I propodus width	
				Gnathopod I propodus diagonal	
				Gnathopod II propodus length	
				Gnathopod II propodus width	
				Gnathopod II propodus diagonal	
D	Spines	Defensive	Defensive, anti-predatory trait in some species.	Presence/absence of spines	^29,38,39^
E	Uropods	Presumably involved in locomotion	Sexually dimorphic trait in some species.	Uropod I basis length	^37,40^
				Uropod I endopodite length	
				Uropod I exopodite length	
				Uropod III exopodite proximal article length	
				Uropod III exopodite distal article length	
	Number and size of eggs	Reproductive	Egg number per single brood affects overall fecundity of a female. Egg size corresponds to yolk volume available to the developing embryo.	Egg number	^26^
				Egg diameter	

**Fig. 1:**
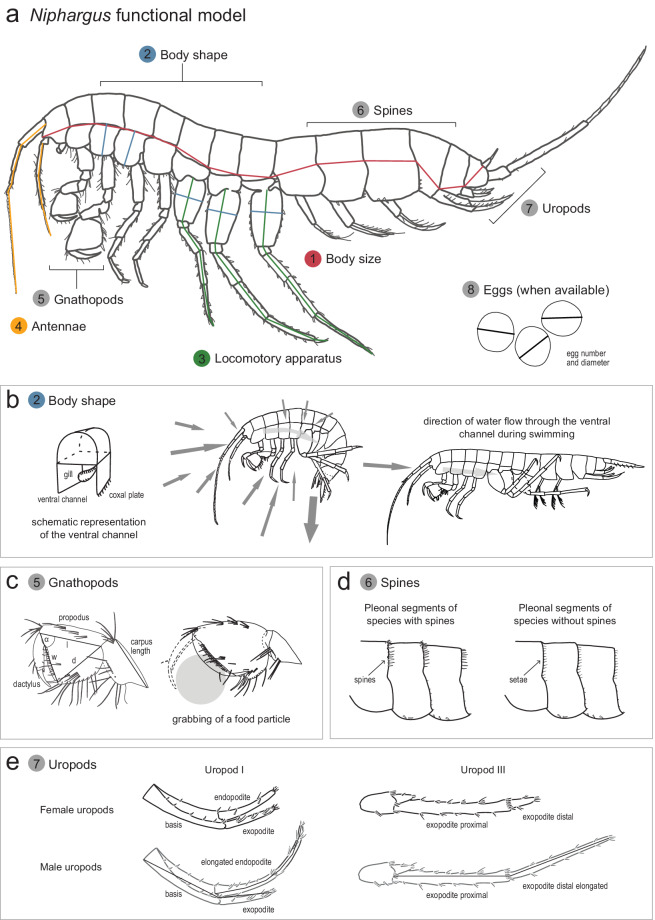
*Niphargus* functional model. An example of *Niphargus* specimen (**a**) and measured morphological functional traits, excluding egg diameter. Lines represent measurements of traits according to the landmarks^37^. Panels b–e detailly represent four functional traits. Measurements of gnathopods and uropods are shown on panes c and e, respectively.

We acquired 27 morphological measurements collectively representing eight morphological functional traits: body size, body shape, locomotory apparatus, antennae, gnathopods, defense spines, uropods, and number and size of eggs. *Body size* is a fundamental trait related to species’ biology. It affects the organisms’ metabolic rate, ability to disperse, their locomotion, and microhabitat selection^24,32^. Similarly to body size, *body shape* relates to locomotion and microhabitat selection^24,27^, but also determines the shape of the ventral channel, which controls the velocity of water flow bringing oxygen to the gills and creating propulsion for movement^33^ (Fig. 1b). In females, the shape of the ventral channel is also connected to the space available for marsupium^26^.

We further measured morphological traits for *locomotory*, *sensory*, *feeding*, and *defensive* functions. Pereopod length (trunk appendage, see Fig. 1) relates to movement speed in *Niphargus*. Species with longer pereopods move faster than species with shorter pereopods^27^. Antennae play an important role in amphipod chemoreception^33^. To account for sensory function, we measured the length of both pairs of antennae. Further, the first two pairs of amphipod pereopods, the gnathopods, are modified for grabbing food particles, guarding females, and grooming^34–36^. In *Niphargus*, their size and shape relates to feeding habits^25,28^. Species with larger gnathopods tend to occupy higher trophic positions and feed as predators, whereas species with smaller gnathopods occupy lower trophic positions and feed as detritivores^28^. Gnathopod shape determines how broadly the last two articles, propodus and dactylus, open, and defines the maximum size of the food particle that the animal can grab^37^ (Fig. 1c). The reach depends on so-called palmar angle of propodus. Propodi with smaller angles enable grabbing relatively larger food particles and are generally more triangle-shaped, whereas propodi with larger angles enable grabbing of only smaller food particles and are square-shaped. Finally, amphipods may express anti-predatory traits^38,39^. In *Niphargus*, pleonal spines play a defensive role and are present in several species^29^ (Fig. 1d).

Besides abovementioned functional traits, we also included data specific for some species and individuals related to sexual selection and fecundity, respectively. Several species of *Niphargus* exhibit sexual dimorphism. Previous studies have identified sexually dimorphic traits, including body size and relative lengths of abdominal appendages, the uropods^40^. In species exhibiting sexual dimorphism, males are larger than females and have longer uropods^40^ (Fig. 1e), which are presumably also involved in locomotion. Lastly, female body size and shape relates to brood size, with larger amphipods having more eggs^26,41^. We counted number of eggs and measured their diameter (whole clutch except when some of the eggs were damaged) in females that carried eggs at the time of their collection.

### Samples acquisition and ecological characterization

**Fig. 2:**
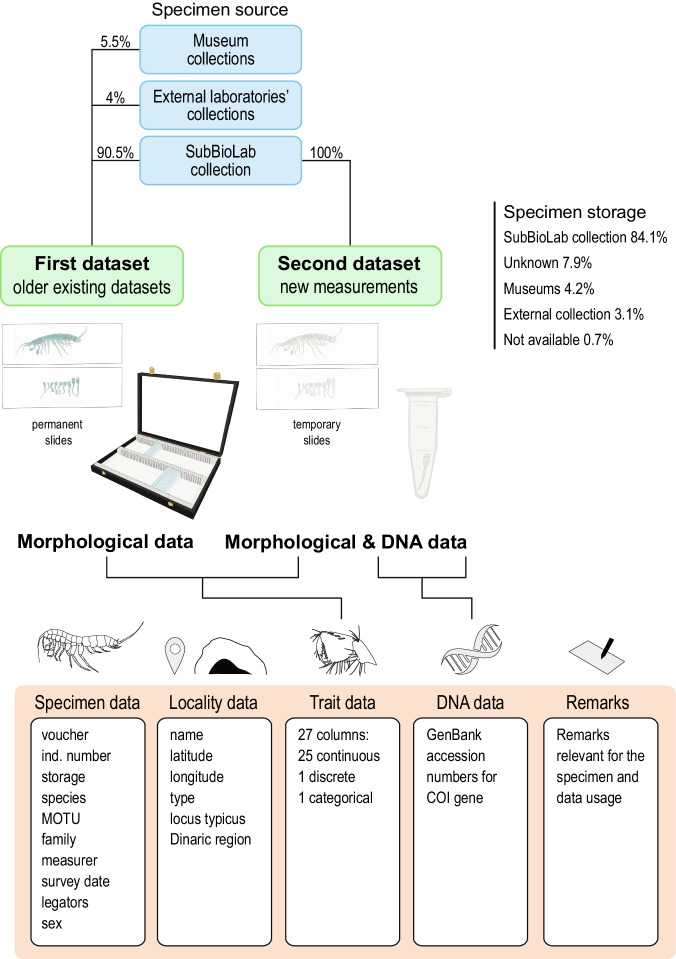
Overview of data collection and dataset structure for both families, Niphargidae and Typhlogammaridae. Most of the specimen and locality data originates from SubBioDB^48^.

**Fig. 3:**
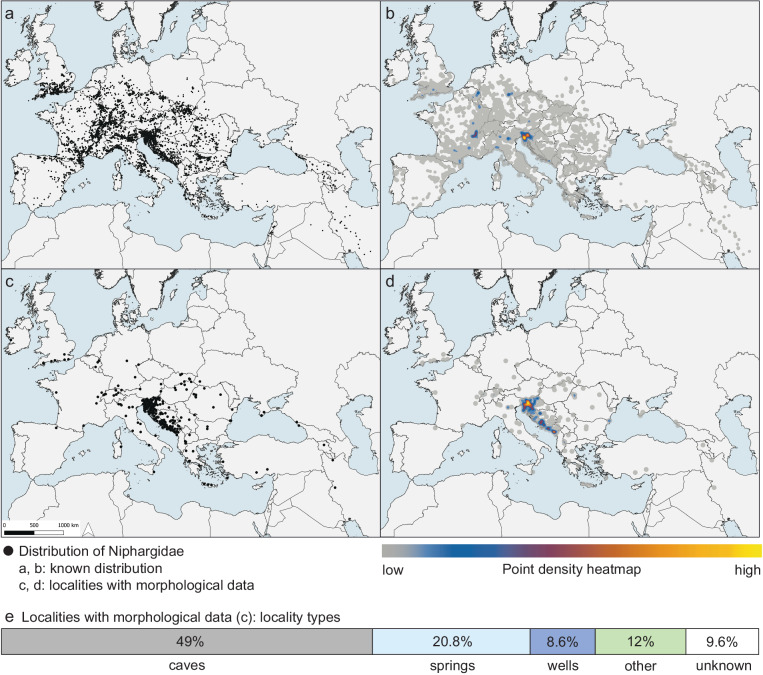
Distribution of Niphargidae. (**a** and **b**) Currently known distribution of Niphargidae^21,46,48^ and localities with morphological data **(c,d)**. Panels a and c show individual data points, and panels b and d the same data as a heatmap. The highest density of Niphargidae localities is in Slovenia (panel b), whereas density of localities with morphological data extends from Slovenia across Dinarides (panel d). Panel e summarizes types of the localities with morphological data. Type “other” includes different freshwater habitats (interstitial, brook, river, lake, puddle, wetland) and artificial habitats (tunnel, ditch, borehole).

Most of the samples were collected during field work of researchers from the Department of Biology, Biotechnical Faculty, University of Ljubljana, between 1950s and 2022 (Fig. 2). Samples were collected from different aquatic habitats, such as springs, caves, brooks and river interstitial, and artificial habitats, such as wells and tunnels, using adequate sampling techniques according to habitat type. Animals were mostly collected using different water nets, but also with the usage of other methods, as e.g. Karaman-Chapuis and Bou-Rouch sondes^42^ or water traps. The sampling has been most intensive in Slovenia and Dinarides, a karstic massif in the SE Europe (Fig. 3), for two reasons. First, Dinaric subterranean habitats have traditionally been at focus of many Slovenian and foreign researchers due to extremely high diversity of subterranean species^43,44^. Second, *Niphargus* diversity peaks in the Dinarides^45,46^, and sampling campaigns were often dedicated to collection of *Niphargus* for different taxonomic, ecological, and evolutionary studies.

Many ecological studies benefit from inclusion of species’ preferred habitat. Groundwater habitats are heterogenous and differ in environmental parameters, such as isolation from surface, connectivity, pore size, water flow velocity, and chemistry, all of which affect different aspects of species’ ecology^45,47^. A habitat preference can be assigned to a species from multiple records and analysis where species most frequently occurs. We included locality type where an individual was collected whenever possible to allow inference of species habitat and ecological preferences based on occurrence frequencies in different locality types. We acknowledge that epigean freshwater and groundwater habitats may be classified in a variety of ways using different parameters as a key criteria. In this dataset, we followed general locality types as in SubBioDB, an internal database on subterranean fauna^48^ (e.g. cave, spring, river interstitial), which do not include microhabitat details (e.g. cave puddles, cave streams) and are not restricted to only groundwater habitats. This way, we avoided potential drawbacks of microhabitats due to different levels of accuracy, and at the same time enabled inclusion of other amphipod taxa from other freshwater habitats.

### Specimen selection, preparation, and measurement

The dataset consists of two sets of morphological measurements. The first set gathers data obtained from older samples and existing microscopic slides. Some of these data were already published, but scattered across many publications. We compiled and unified these individual published and internal datasets for the whole distribution range of Niphargidae. The second set represents morphological data acquired in recent years to cover as many species and undescribed species (discovered through the usage of molecular methods, hereafter referred to as MOTUs: Molecular Operational Taxonomic Units) as possible following the latest available phylogeny^49^. In this second set, we focused on the most well-sampled area, Slovenia and the Dinarides, due to the availability of the material. In the second set, we selected the individuals with best-preserved morphology, and preferentially used those that already had available DNA sequences or additionally sequenced them in the case of uncertain morphological diagnosis.

In the first set, the collected individuals were mounted on microscopic slides. They were treated in a hot solution of 10% KOH, rinsed with HCl and distilled water, and stained using different pigments. After treatment, they were dissected and put on the slides in a gelatine glycerol medium (Merck) and measured under stereomicroscope. Contrary to the first set of measurements, animals of the second set were not mounted on permanent microscopic slides, but rather prepared temporarily to preserve the animal for other analyses and DNA extraction. These animals were stored in collection in 96% ethanol at −20 °C. Prior measurements, we transferred them to glycerol for 1–2 days and dissected them. We put them on the slides without any additional treatment by using glycerol as a medium, measured them under stereomicroscope, and stored them back in 96% ethanol at −20 °C. The reasons behind such preservation of the animals are their rarity and effort associated with obtaining additional samples for other analyses. We assessed potential differences and errors due to different measurement techniques, which turned out as negligible.

When measuring morphological traits, we followed the standard landmarks for morphological measurements of *Niphargus*^37^ (Fig. 1). We used two stereomicroscopes with mounted digital cameras and corresponding software: Olympus SZX9 coupled with ColorViewIII camera and cellSens Entry software, and Leica M165C coupled with Leica Flexacam C1 camera and LASX software. The magnification used depended on the specimen size. For specimens measured within the second set, images are stored and measurements repeatable directly on the images. They can also be exported in optional formats and resolutions of up to 4000 × 3000 pixels.

### The identifier issue resolved: individual *versus* MOTU *versus* species

The dataset is constructed at an individual level that allows wide reusability of the data and analytic flexibility. It also establishes a stable link between an individual and its measurable attributes, including morphometric data, distribution, DNA sequence, and ecology^50^. The dataset allows easy addition of new data, making it volatile and reusable in different analytic frameworks. With addition of new samples, morphometric data can be reused, and, for example, estimations of the mean trait value and its variation can be refined. Similarly, addition of new individual-level ecological data may provide additional insights into species ecology. Such refinements would not be possible if data were entered as mean values of the populations or species. Hence, the addition of records will make the dataset reusable for analyses at three hierarchical levels: among-individuals, between-populations, and between-species.

The main reason for individual-level organization of the dataset is the inherent nature of taxonomy, i.e. species hypothesis^51,52^. Addition of new data in the dataset may yield new species hypotheses, i.e., an individual can be assigned to another species after revision. If addition of new records yields a modified taxonomic structure of the dataset, e.g., species A is split into species A and B, individuals with barcodes are reassigned to new species and biological attributes automatically follow the revised taxonomic structure without needing a revision^51^. This flexibility does not hamper analyses relying on population or species level, as we provide customized scripts for estimation of mean values, or population-level traits, such as sexual dimorphism, as discussed under the Usage Notes.

Individuals in our dataset are labelled using voucher system from the SubBioDB^48^. In separate columns, we assigned MOTU and/or species names to each individual (except rare cases, see Data Records). To keep the link with previous publications, we followed already established labelling system of MOTU names^46,49^. This three label-system might seem redundant, however, we find it useful to comply with requirements of contemporary ecological and evolutionary research that relies on MOTUs, biological conservation that depends on Linnean taxonomy, and technical management of the dataset, where individuals as biological attribute bearers can be reassigned to revised taxonomic structure at minimal effort and minimal chance for introduction of errors or data loss^50,51,53^.

## Data Records

The dataset is available for download from figshare^54^ as a Microsoft Excel (.xlsx) file. The first sheet, named Data, contains all raw data, and the second sheet, named Description, contains descriptions of columns and values similarly as in Supplementary Table 1.

The dataset includes data related to an individual and data related to a locality where it was collected (Fig. 2). The first 16 columns summarize metadata about the measured individual and locality. They include voucher and/or individual number, species and MOTU identities, family, storage, measurer, survey date, legators, and sex, and columns providing data on the locality include name, geographical coordinates, and locality type. Additional two columns carry information on whether the locality is a type locality of a species and whether it lies within Dinaric region or not. With the extension of the dataset, especially by addition of records outside the Dinaric region, the last column can be transformed into “biogeographical region”.

The morphological data are contained in 27 columns and include 25 columns with morphometric (continuous) functional trait data and two columns with discrete or categorical morphological functional trait data. Lastly, we included information on COI sequences and remarks important for the usage of the dataset. An overview of the column contents is given in Supplementary Table 1.

The dataset includes measures of 1123 individuals. They belong to two families (1092 to Niphargidae and 31 to Typhlogammaridae), 180 species and 314 MOTUs. We identified species based either on morphological data or DNA sequence, while we assigned MOTUs to individuals where there was at least one sequence from a locality where individuals were sampled. For an additional 51 individuals, we assigned MOTUs even less stringently, when the measured individuals were from type locality or when morphology was studied in detail, although not connected with a DNA sequence. We entered this information accordingly in the “remarks” column. In the case of undescribed species, we assigned “sp.” as a species name, and entered MOTU information in the MOTU column. Only five individuals remain without both species and MOTU identities. This less stringent assignment of MOTUs increases sample sizes per MOTU in downstream analyses and secures reusability of the available data.

**Fig. 4:**
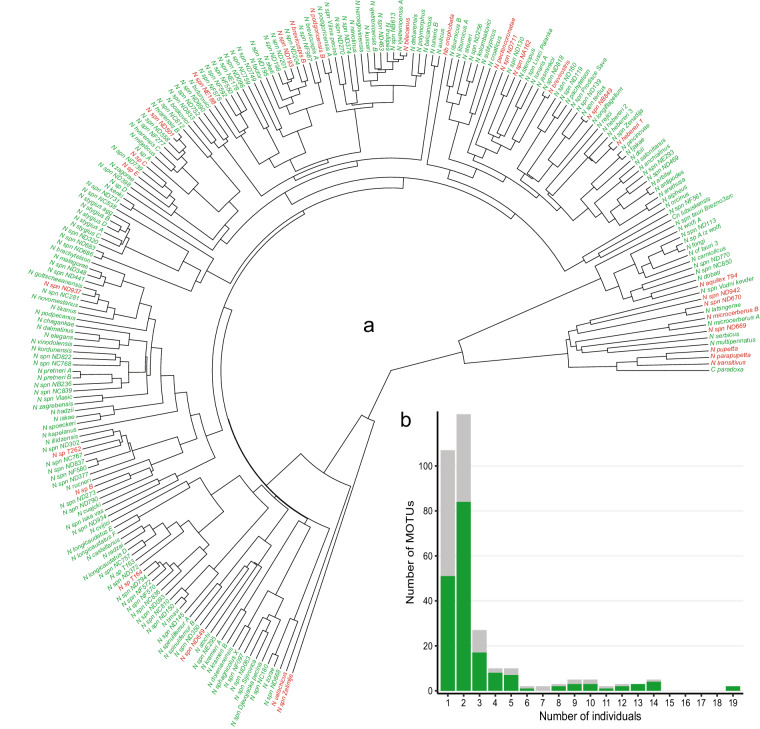
Dinaric Niphargidae phylogeny and overview of number of individuals measured per MOTU. (**a**) phylogenetic tree^49,54^ pruned to MOTUs distributed in the Dinaric region. Tips colored according to data availability (green: available morphological data, red: missing morphological data). In the most species-rich and sampled region, the share of MOTUs with missing data is low. (**b**) Numbers of individuals measured per MOTU colored by distribution (grey: whole dataset, green: limited to MOTUs distributed in the Dinaric region). Most MOTUs are represented with at least two specimens.

When obtaining data in the second set of measurements (see previous section and Fig. 2), we aimed to measure at least two adult specimens per MOTU when enough material was available. This approach captured at least some intra-MOTU variability, but also enabled more accurate estimates of missing data (e.g. when one specimen is missing one trait and the second one another trait, both can be imputed by using data from the closest specimen in the phylogeny; see Usage notes). An overview of number of specimens per MOTU and number of MOTUs is given in Fig. 4.

The specimens included in the dataset were collected at 389 different localities spread across the whole range of both families^23^, but with most of the localities clustered in Slovenia and in the Dinaric region (Fig. 3a,b). Most of the localities are by their type caves (49.4%) followed by springs (20.6%), and wells (8.5%). 11.8% and 9.8% of the localities are of other or unknown types, respectively (locality types of Niphargidae shown on Fig. 3e).

## Technical Validation

We constructed as detailed and as complete functional trait dataset for subterranean amphipods as possible in terms of reliable species and MOTU identification, measurement accuracy and completeness, spatial coverage, and phylogenetic coverage. The link between the morphological trait and its function was tested in eight independent studies (see Table 1 for references).

Phenotypic identity of the specimens was assessed by experts (CF). When species identification was uncertain or in the case of cryptic species, we also examined genetic identity by sequencing the COI or multiple genes. Nine-hundred and five (80.6%) specimens have MOTU identification. A large part of the dataset thus provides the most detailed phenotypic and genotypic identity of the specimens.

Morphometric data were obtained mostly by two researchers (CF, first dataset, and EP, second dataset) with minor contributions of others, hence minimizing the error related to individual measuring differences. As body length influences all other continuous and discrete traits and possible downstream analyses which would account for individual’s body length, we measured it three times and included the mean value of the three measurements to the dataset.

Among Niphargidae, the amount of missing data does not exceed 14% per continuous trait measurable in both sexes, except for uropod measurements (41–50%). Most of other continuous traits have less than 5% of missing data (13 traits out of 19), while 6 have 5–14%. We provide an example workflow to deal with missing data in Usage Notes. The related R code is available from figshare^54^.

The dataset covers well both currently known spatial distribution of Niphargidae throughout the Western Palearctic and the latest phylogeny. The largest share of the data is available for the Dinaric region and Slovenia (Fig. 3c,d). Phylogeny includes 561 MOTUs, and 300 (53,5%) out of those also have morphological data. When pruned to the Dinaric region (215 MOTUs), which represented the focal area in obtaining the second set of measurements, the share of MOTUs with morphological data increases to 86% (Fig. 4a).

To validate the possibility of wider usage of selected traits across different groundwater amphipod taxa, we additionally measured individuals belonging to another groundwater amphipod family present in Europe and distributed in the Dinarides, Typhlogammaridae. We measured specimens belonging to three genera: *Metohia, Accubogammarus*, and *Typhlogammarus*. We proved that measurement of the functional traits included in our dataset is applicable beyond Niphargidae, which opens a possibility for future broadening of the dataset.

## Usage Notes

Usage Notes refer to the usage of data for the family Niphargidae and were so far not applied in Typhlogammaridae. For all sections, we provide R code to repeat the calculations and imputation of missing data. It is available for download from figshare^54^. The R script was written in R version 4. 2. 2^55^ using R Studio version 2022.12.0.353^56^ and packages phytools 1.2-0^57^, ape 5.6-2^58^, PVR 0.3^59^ and missForest 1.5^60^.

### Quantification of gnathopod size and shape

Gnathopod size and shape relate to *Niphargus*’ feeding habits and can be quantified using three raw measurements of the sixth gnathopod article, propodus^25,28^. Gnathopod size is quantified as gnathopod perimeter, a sum of propodus length (pl), width (pw), and diagonal (pd) (marked l, w, d in Fig. 1c; Eq. (1)). Gnathopod shape is defined by the angle α between the propodus length and width (Fig. 1c). This angle can be retrieved using the same three raw measurements, propodus length, width, and diagonal, and cosine theorem (Eq. (2)).

1 $${\rm{G}}{\rm{n}}{\rm{a}}{\rm{t}}{\rm{h}}{\rm{o}}{\rm{p}}{\rm{o}}{\rm{d}}\,{\rm{p}}{\rm{e}}{\rm{r}}{\rm{i}}{\rm{m}}{\rm{e}}{\rm{t}}{\rm{e}}{\rm{r}}={\rm{p}}{\rm{l}}+{\rm{p}}{\rm{w}}+{\rm{p}}{\rm{d}}$$

2 $$\cos \alpha =\frac{{{\rm{pw}}}^{2}+{{\rm{pl}}}^{2}-{{\rm{pd}}}^{2}}{2\cdot {\rm{pl}}\cdot {\rm{pw}}}$$

### Sexually dimorphic traits

The dataset is structured on an individual level, and as such does not provide population-, MOTU-, or species-level traits, as e.g. the presence of sexual dimorphism, which occurs in several Niphargidae species^40^. However, this information can be obtained for 75 species included in the dataset using measurements of body length, uropod I endopodite and exopodite, and uropod III proximal and distal articles of exopodite (Fig. 1e). Males of sexually dimorphic species are larger than females and have relatively longer uropod I endopodite and exopodite of uropod III. To assess differences between males and females, the length of uropod I endopodite can be compared against the length of uropod I exopodite, while the length of uropod III exopodite distal article can be compared against the length of uropod III exopodite proximal article. The ratios of uropod I endopodite: uropod I exopodite and uropod III exopodite distal article: uropod III exopodite proximal article are larger in males.

### Imputation of missing data

Trait datasets often contain missing values. To some extent, this can be avoided during data collection, but not entirely. Missing values can cause issues in downstream analyses and can be handled in many ways. The simplest and most rigid approach is to remove entries with missing data, but this may heavily affect the size and strength of the dataset. On the other hand, missing values can be replaced with imputed values through several different computational approaches which differ mostly in computational background and ability to incorporate phylogenetic information^61,62^. As our dataset contains missing values, we here provide an example workflow for their imputation.

We imputed missing values similarly as in Penone *et al*.^61^ and Debastiani *et al*.^63^ using missForest package^60^ which uses Random Forest algorithms. We used both trait data and phylogenetic information to impute missing values, as the latter usually improves estimations^61^. We imputed missing values in continuous traits, excluding uropod and egg traits which were not measured in many individuals, keeping the traits on a level of single individual. To incorporate phylogenetic information, we followed the approach of Penone *et al*.^61^ and Debastiani *et al*.^63^ by first transforming phylogenetic distance matrix into orthogonal vectors. We selected the number of eigenvectors (first 40) and added them to the trait dataset.

Imputed missing values should be handled carefully and checked for their accuracy^62^. We evaluated the accuracy of estimated values in two ways. First, we checked the out-of-box imputation errors (NRMSE, normalized root mean squared error) returned by the Random Forest algorithm for each trait separately. Second, we calculated the ratios between trait value and body length for observed and imputed data for each MOTU and summarized them in a table where they can be easily compared and checked for any outliers or errors.

### Supplementary information

Supplementary Table 1

## Data Availability

The R code used for summaries of the Data Records and for creating the plots in Fig. 4, as well as the R code for the analyses described in the Usage Notes are available for download from figshare^54^. We did not use any other custom code during data collection.
